# GeneTerpret: a customizable multilayer approach to genomic variant prioritization and interpretation

**DOI:** 10.1186/s12920-022-01166-3

**Published:** 2022-02-18

**Authors:** Roozbeh Manshaei, Sean DeLong, Veronica Andric, Esha Joshi, John B. A. Okello, Priya Dhir, Cherith Somerville, Kirsten M. Farncombe, Kelsey Kalbfleisch, Rebekah K. Jobling, Stephen W. Scherer, Raymond H. Kim, S. Mohsen Hosseini

**Affiliations:** 1 Ted Rogers Centre for Heart Research, Cardiac Genome Clinic, The Hospital for Sick Children, Toronto, ON Canada; 2 Department of Electrical Engineering and Computer Science, York University, Toronto, ON Canada; 3 Department of Molecular Genetics, Faculty of Medicine, University of Toronto, Toronto, ON Canada; 4 MIT Sloan School of Management, Massachusetts Institute of Technology, 100 Main Street, Cambridge, MA 02142 USA; 5 Faculty of Medicine, University of Toronto, Toronto, ON M5S1A8 Canada; 6 Ted Rogers Centre for Heart Research, Toronto General Hospital Research Institute, University Health Network, Toronto, ON Canada; 7 Genome Diagnostics, Department of Pediatric Laboratory Medicine, The Hospital for Sick Children, Toronto, ON Canada; 8 The Centre for Applied Genomics, The Hospital for Sick Children, Toronto, ON Canada; 9 Program in Genetics and Genome Biology, The Hospital for Sick Children, Toronto, ON Canada; 10 Centre for Genetic Medicine, The Hospital for Sick Children, Toronto, ON Canada; 11 Department of Molecular Genetics, University of Toronto, Toronto, ON Canada; 12 Division of Clinical and Metabolic Genetics, The Hospital for Sick Children, Toronto, ON Canada; 13 Fred A. Litwin Family Centre in Genetic Medicine, University Health Network, Department of Medicine, University of Toronto, Toronto, ON Canada; 14 Department of Pathology, The University of Texas MD Anderson Cancer Center, Houston, TX USA

**Keywords:** Genome interpretation, Genomic variants, Genotype–phenotype correlation, Disease gene validity, Variant pathogenicity, Causative variants, Gene prioritization, Bioinformatic application

## Abstract

**Background:**

Variant interpretation is the main bottleneck in medical genomic sequencing efforts. This usually involves genome analysts manually searching through a multitude of independent databases, often with the aid of several, mostly independent, computational tools. To streamline variant interpretation, we developed the *GeneTerpret* platform which collates data from current interpretation tools and databases, and applies a phenotype-driven query to categorize the variants identified in the genome(s). The platform assigns quantitative validity scores to genes by query and assembly of the genotype–phenotype data, sequence homology, molecular interactions, expression data, and animal models. It also uses the American College of Medical Genetics and Genomics (ACMG) criteria to categorize variants into five tiers of pathogenicity. The final output is a prioritized list of potentially causal variants/genes.

**Results:**

We tested *GeneTerpret* by comparing its performance to expert-curated genes (ClinGen’s gene-validity database) and variant pathogenicity reports (DECIPHER database). Output from GeneTerpret was 97.2% and 83.5% concordant with the expert-curated sources, respectively. Additionally, similar concordance was observed when *GeneTerpret’s* performance was compared with our internal expert-interpreted clinical datasets.

**Conclusions:**

*GeneTerpret* is a flexible platform designed to streamline the genome interpretation process, through a unique interface, with improved ease, speed and accuracy. This modular and customizable system allows the user to tailor the component-programs in the analysis process to their preference. *GeneTerpret* is available online at https://geneterpret.com.

**Supplementary Information:**

The online version contains supplementary material available at 10.1186/s12920-022-01166-3.

## Background

Rapid advances in DNA sequencing technologies have enabled the revolutionary use of clinical genomic data to support precision medicine initiatives, improving patient care and medical management. The process of Genomic Variant Interpretation (GVI) aims to identify one or a few medically relevant variants from hundreds of thousands in a genome [1]. To do this accurately, the genomic evidence supporting the association of a candidate gene with a disease of interest (gene-disease validity) and the detrimental effect of a variant on the gene function (variant pathogenicity) must be evaluated. Although several independent computer programs are available to aid GVI, the process routinely requires manual interpretation by a human analyst who leverages expertise, insight and phenotypic knowledge to curate a list of candidate variants. This process is often tedious, repetitive, time-consuming, and may be prone to human errors. Therefore, it is not surprising that discordance exists among germline variant classifications across laboratories/groups, diseases, and variant types [2]. Part of the discordance is due to different technologies and variant interpretation pipelines utilized. Accordingly, a unifying platform for GVI is needed to help standardise the process and outcomes.

The currently available GVI platforms and tools take various approaches to provide different levels of support for genome interpretation. However, these usually do not present all the required tools in a comprehensive interactive package, lack proper validation, or use limited resources in their classification/interpretation. A growing number of these tools have been bundled into commercial or free packages to aid in genome interpretations for rare Mendelian disorders. Some of the most popular publicly available, web-based tools to assist genome analysis include GeneTalk [3], eXtasy [4], Phen-Gen [5], Exomiser [6], OVA [7], QueryOR [8], Variant Ranker [9], Mutation Distiller [10], and VarFish [11]. A common feature among these GVI tools is their ability to integrate user-defined phenotypic information into their variant filtering and prioritization framework. These platforms provide either variant pathogenicity assessment or gene-disease validity evaluation, or a combination of both in rare cases. However, they rarely provide a unified streamlined all-in-one platform for genome interpretation. Most of these platforms do not provide comprehensive curation of the various levels of evidence, or appropriate application of the ACMG criteria. Moreover, these platforms either lack the flexibility to provide an iterative reweighting workspace for the user to define what evidence should be considered, or go overboard by providing tens of filters that a user needs to adjust without having an appropriate point of reference (refer to Additional file 2: Table S1).

In an effort to facilitate genome interpretation by presenting a unified all-in-one platform for the average genome analyst, we have developed *GeneTerpret*, a customizable GVI platform, and visual analytics tool that accelerates the prioritization of genomic variants with an easy interface for expert interaction. The platform considers both phenotypic and genomic information to produce and prioritize a list of putative medically relevant variants. The platform can accurately analyze genomes from singletons, trios, or entire cohorts, and extract a significantly more manageable candidate-gene list for a human analyst to review. Overall, *GeneTerpret* improves the GVI process by increasing the speed, and therefore reducing associated costs, while providing the analyst with the freedom to customize the platform’s parameters, filters and outputs. Platforms like *GeneTerpret* can ultimately help to improve accuracy and reduce the inter-lab variability in variant interpretation.

## Implementation

### *GeneTerpret* workflow and implementation

**Fig. 1:**
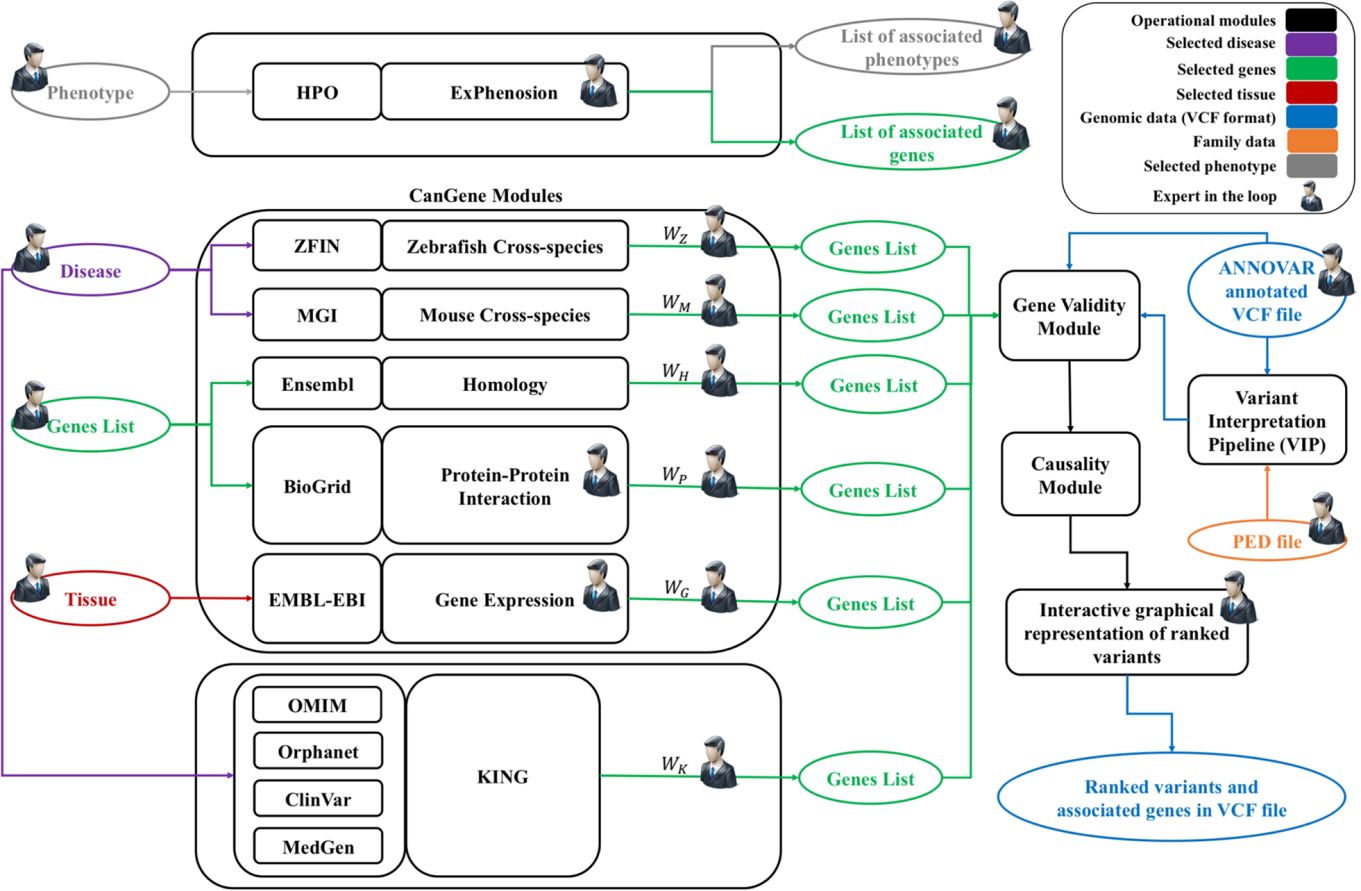
*GeneTerpret* workflow. The figure depicts the modules, their feeding databases, acceptable inputs, and the flow of information in the workflow. The main modules feed into the gene validity, *VIP*, and causality modules. Three sets of modules are available within *GeneTerpret* for gene validity exploration; (1) ExPhenosion module—accepts the phenotype as input; the number of super-classes to walk up can be customized; and outputs the connected phenotypes and their associated genes. This module works independently from other developed modules to extract the connected phenotypes to the selected phenotype and allow the analyst to explore the genes associated with related phenotypes; (2) CanGene modules—generate a list of candidate genes by compiling various types of evidence. The cross-species (zebrafish and mouse) modules accept the disease(s) by its/their MONDO ID(s) as the input(s) and generate a list of genes that their orthologue is associated with similar disease in animal models by checking the related databases. The Homology and Protein–Protein Interaction modules accept a list of known genes for a phenotype (if it is available); the Homology module returns the homologous genes (paralogues) to the genes in the known gene list. The Protein–Protein interaction module takes a similar approach to generate a list of genes that interact with the known disease genes. The analyst can select the number of interaction neighborhood levels (such as level-1, level-2, etc.) desired for this interpretation. The Gene Expression module accepts a list of relevant tissues as input and outputs the list of genes expressed in the selected tissue based on the expression cut-off threshold which is set by the analyst; (3) *KING* module—accepts a disease(s) (MONDO ID(s)) as the input and then outputs a list of genes associated with the said disease based on evidence obtained from Orphanet, OMIM, ClinVar, and MedGen databases. The validity module accepts the generated gene lists from the modules CanGene and *KING*, as well as ANNOVAR, annotated VCF file or the output of VIP module as an input. The output file is the VCF file including validity scores. VIP module has been developed based on ACMG guidelines [16]. This module annotates the variants with pathogenicity terms (PVS1, PS1, etc.) and justifies the assigned terms. The causality module integrates the output of validity and VIP modules and ranks the variants based on the number of evidence extracted from validity modules and pathogenicity terms from VIP. Simultaneously, an interactive graphical representation of the variants is generated which allows the analyst to select the desired variants by using a LASSO filter

The *GeneTerpret* platform execution is modular and customizable, allowing the user to generate candidate gene lists based on different inputs and parameters, such as specific tissue type, phenotype, and known gene(s). It accepts genotype data and family information in Variant Call Format (VCF) and Pedigree (PED) file formats. The outputs of *GeneTerpret* analysis can be a more refined list of genes, their associated phenotype(s), and VCF files for further consideration. An overview of the *GeneTerpret* platform workflow is summarized in Fig. 1. More details on the backend and web implementation are presented in the Additional file 1: S1 section. In brief, the workspace area is accessible through a Graphic User Interface (GUI) and acts as a prototypical canvas upon which phases of query and data processing are performed. Each entry is represented as a node that can be flexibly added or removed to achieve a user-desired analysis scheme. Nodes can be intuitively connected to data or modules of compatible inputs and output to allow the flow of data, with the platform and associated algorithms executing the corresponding module functions in the backend. This way, the user can quickly apply these complex functions to data, triggering the execution of the backend functionalities. Once the results are ready, the user can download the compressed file of prioritized genes for review, while the inputted data and settings from a recent analysis session remain open for the user to re-customize and fine-tune the analysis. Additional file 4: Figure S1 shows an example scenario of the GUI during the implementation of the platform workflow.

### *GeneTerpret* modules and functions

#### Generating and querying known and candidate gene lists and exploring phenotype associations

To establish gene-disease validity in *GeneTerpret*, the general interpretation workflow consists of three modules that extract the phenotype terms, their associated genes, and their candidate genes. The first module, Known INvolved Genes (*KING*), outputs a list of genes associated with a particular phenotype(s), with solid evidence of support from *OMIM* [12], *Orphanet* [13], *MedGen* [14], and *ClinVar* [15]. The second module, Expanded Phenotype Exploration (ExPhenosion), uses the Human Phenotype Ontology (*HPO*) hierarchy of phenotype [16] to produce a list of genes associated with a particular phenotype, (e.g. *Tetralogy of Fallot*), including superclass terms which is the broader phenotype category for a specific HPO term (e.g. *Conotruncal defect*). The expanded phenotypes and associated genes from this module can be fed into other modules to increase the scope of evidence generation. The third module, Candidate Genes (*CanGene*), produces a list of candidate genes for a given phenotype by collecting various pieces of biological evidence from many relevant databases (Table 1). The details of each module and databases used by *GeneTerpret* are described in S2 section and Additional file 1: Table S2.

**Table 1:** Databases used by operational modules in the *GeneTerpret* Platform

Module	Task	Databases
ExPhenosion	Identifies genes associated with the selected phenotype(s) and its/their superclass phenotypes	Human Phenotype Ontology (HPO) Medical Subject Headings (MeSH)
CanGene		
Cross-species: Mouse	Identifies candidate genes that cause a “similar” phenotype in a mouse model	Mouse Genome Informatics (MGI)
Cross-species: Zebrafish	Identifies candidate genes that cause a “similar” phenotype in the zebrafish model	The Monarch Initiative
Homology	Identifies candidate genes homologous to known disease genes for a phenotype	Ensembl
Protein–Protein interaction	Identifies candidate genes/proteins that physically interact with known disease genes/proteins based on human studies	The Biological General Repository for Interaction Datasets (BioGRID)
Gene Expression	Identifies candidate genes expressed in the affected tissue	EMBL-EBI Expression Atlas
Known INvolved Genes (*KING*)	Identifies known genes for a selected phenotype	Online Mendelian Inheritance in Man (OMIM) Orphanet NCBI MedGen NCBI ClinVar
Gene Validity	Calculates validity scores for each gene by examining the strength of the evidence supporting a gene-disease relationship obtained from the above modules	N/A
Variant Interpretation Program (*VIP*)	Classifies variants based on their pathogenicity following the criteria proposed by the American College of Medical Geneticists (ACMG)	ClinGen Dosage Sensitivity Map Decipher haploinsufficiency predictions ExAC pLI score ClinVar The NHGRI-EBI Catalog of published GWAS Pfam clans Weil et al. 2017 [25]
Causality	Graphical visualization of the distribution of prioritized variants across the five classifications of pathogenicity	*GeneTerpret* GUI

#### Gene validity module—integration of validity terms

The gene validity module is used to quantify the strength of evidence that supports a gene-disease relationship. This module consolidates output gene lists from *CanGene*, *ExPhenosion*, and *KING* modules (see gene validity module architecture in Additional file 5: Figure S2), and appends a score for each gene based on the number of times it appears in the output of the modules, the user-assigned weights for each module, and the user-defined thresholds. We recommend that *KING* output be considered as strong evidence (known genes), as it is based on published genes associated with human phenotypes in the four common medical genetics databases, while the other outputs can be treated as limited evidence (candidate gene). The acceptable inputs for gene validity modules are (a) gene lists obtained from any of *CanGene*, or *KING* module, (b) uploaded gene-list, or (c) uploaded VCF file. The module output provides a new, annotated VCF file with added column(s) showing the weight of evidence for each gene from the list previously generated from each selected module. For each gene, a validity score that summarizes all evidence is also provided in the output. It is important to note that the module parameters (such as the thresholds and weights) set by the analyst will impact the validity scores produced.

#### Variant interpretation program (*VIP*) module—determining variant pathogenicity

The Variant Interpretation Program (*VIP*) module establishes and appends pathogenicity calls to variants from a given VCF file. The internal structure of this module has been shown in Additional file 6: Figure S3, and the respective databases used in this module are presented in the Additional file 1: Table S2. This module accepts a VCF file in *ANNOVAR* annotation format (Additional file 1: Table S3), and where family history is available, a combination of PED and VCF files as its input to achieve trio analysis (example in Additional file 1: Table S4). The module outputs a set of new annotated VCF files, each with new columns added, showing ACMG pathogenicity classification for each variant, the ACMG criteria invoked, and the justification for arriving at a given classification. Overall, for each sample/trio/cohort analyzed, three VCF files are created; the first contains only de novo variants (if sufficient data provided), the second lists only pathogenic and likely pathogenic variants, and the third lists all variants with pathogenicity classification. It is important to emphasize that variant pathogenicity classifications from *VIP* do not intend to conclusively indicate a variant’s clinical significance. The variant classifications from *VIP* are merely an algorithmic, non-statistical evaluation of pathogenicity based on thresholds defined in the ACMG guidelines for each variant, and hence do not mean the variant in question is conclusively pathogenic in a particular patient for the phenotype under consideration. Further details of the considered ACMG guidelines [17] and their implementation can be found in Additional file 1: Table S5.

#### Causality module—visualization of the interpreted genomic variants

Where there are many prioritized variants outputted from the *GeneTerpret VIP* module, we realized that a tool for the proper visualization of these variants would be helpful. Therefore, we developed the causality module which uses the output of the *VIP* and plots the variants across the predicted pathogenicity categories against the clinical validity scores of pertinent genes. This module is particularly helpful for visualizing prioritized variants when a high yield of prioritized variants is obtained. Additional file 7: Figure S4 shows a typical graph generated by the causality module which plots the variant distribution in the validity vs. pathogenicity space (Additional file 7: Figure S4(A)). The analyst can further filter the desired variants in this space by using an in-built lasso filtering tool (Additional file 7: Figure S4(B)).

### *GeneTerpret* performance assessment

To assess the performance of *GeneTerpret*, we did a performance comparison assessment in two ways. First, we identified two well-established external resources: ClinGen database [18, 19] for testing clinical validity modules and DECIPHER database [20] for testing the variant pathogenicity module independently. In addition, we used our expert-interpreted internal datasets composed on a Tetralogy of Fallot (TOF) cohort [21] and Cardiac Genome Clinic (CGC) families [22]. All the participants provided informed consent to participate in these studies according to the institutional ethics review board as described in previous publications [21, 22].

## Results

We developed the *GeneTerpret* platform as a bioinformatics tool to facilitate the process of identifying disease-causing variants. Two orthogonal key concepts drive the interpretation of each variant: gene-disease clinical validity, and variant pathogenicity. Gene-disease validity is a qualitative measure of the strength of the evidence supporting the gene-disease relationship, quantifiable according to the ClinGen Gene Curation Project scale [23] as no evidence, limited, moderate, strong, or definitive. For example, one can say SCN5A is “definitively” associated with “Brugada syndrome”, and that a high level of evidence supports the SCN5A-Brugada syndrome relationship [24]. However, we designed *GeneTerpret* not to limit the user to these five categories; instead, the platform allows the user to adjust the weight assigned to each source of evidence to produce a personalized validity factor based on their preferences. The variant pathogenicity output by the platform is a measure of the likelihood of a variant being detrimental to the gene/protein function. Pathogenicity in a clinical setting is expressed on a five-tier classification scale proposed by the ACMG: pathogenic, likely pathogenic, uncertain significance, likely benign, or benign [17]. The causality is defined as the likelihood of a variant explaining the phenotype/disease observed in a patient. So, in *GeneTerpret*, a variant was considered “causal” when it ranked high on both gene-disease validity and variant pathogenicity scales. The details of datasets used and the design of the *GeneTerpret* package are described under methods.

### Validation of *GeneTerpret’s* performance on external data

#### Gene-disease validity module

To validate the performance of the gene-disease validity module, we benchmarked it against the Gene-Disease clinical validity results from ClinGen. These are well established gene-disease associations curated by groups of experts in each field. Of 1082 curation records in the ClinGen Gene Validity curation table (https://search.clinicalgenome.org/kb/gene-validity accessed on September 8, 2020), 715 were classified as “Definitive”, “Strong” or “Moderate” in association with 451 diseases. Running *GeneTerpret*’s *KING* module for these diseases produced a gene list that contained 695 out of 715 genes in the ClinGen gene-validity table (yielding a 97.2% agreement between ClinGen and *KING* module).

#### Performance of VIP

To benchmark the performance of *VIP*, we analyzed the entire DECIPHER dataset and compared the pathogenic/likely pathogenic and benign/likely benign annotations from DECIPHER with results obtained from *VIP*. A summary of the results of *VIP* for all the variants obtained from DECIPHER (8610 variants) is in Table 2. Interestingly, the percentage of variants called to be of uncertain significance were increased (42% in *VIP* vs. 38.6% in DECIPHER) in comparison to a lower percentage of benign/likely benign calls (1.1% vs 2.7%, respectively). Overall, there is high concordance (83.5%) between pathogenic or likely pathogenic calls classified by *VIP* and obtained by DECIPHER.

**Table 2:** *VIP* Interpretation of all variants from DECIPHER

Clinical significance	VIP (Automated Pathogenicity Identifier module)	DECIPHER (Manual Pathogenicity Identifier)	Concordant
Benign	14 (0.1%)	23 (0.3%)	0 (0%)
Likely Benign	81 (0.9%)	211 (2.4%)	9 (0.2%)
Uncertain significance	3633 (42.2%)	3329 (38.6%)	2202 (48.9%)
Likely pathogenic	2692 (31.3%)	2508 (29.1%)	1055 (23.4%)
Pathogenic	2190 (25.4%)	2539 (29.5%)	1240 (27.5%)
Sum of five tiers	8610	8610	4506
Benign or likely benign	95 (1.1%)	234 (2.7%)	9 (0.2%)
Pathogenic or likely pathogenic	4882 (56.7%)	5047 (58.6%)	3764 (83.5%)

### Validation of *GeneTerpret’s* performance on internal data (manually prioritized variants)

To compare the performance of *GeneTerpret’s* variant classification with manual classifications by experienced genome analysts, blinded reinterpretations of two sample datasets were done using the *GeneTerpret* platform. The first set consisted of 10 families, and the second one consisted of 20 individuals, all from our internal database. To rank *GeneTerpret*’s output, we employed a binning system based on scores from the gene-validity module and pathogenicity tier from *VIP* and sorted the variants into four bins. The bins were composed of; (1) pathogenic/likely pathogenic variant in a known (high validity) gene (P/LP KG); (2) pathogenic/likely pathogenic variant in a candidate (moderately valid) gene (P/LP CG); (3) pathogenic/likely pathogenic variant in a novel gene (P/LP NG); and (4) the variant of uncertain significance in a known gene (VUS KG) (Additional file 1: Table S6). Variants in each bin were further ranked based on the validity score of the corresponding genes. Results of validity scores from *GeneTerpret* were then compared with previous interpretations by our experienced geneticists; the latter findings were peer-reviewed and have been published [21].

#### GeneTerpret’s performance in family interpretation

**Fig. 2:**
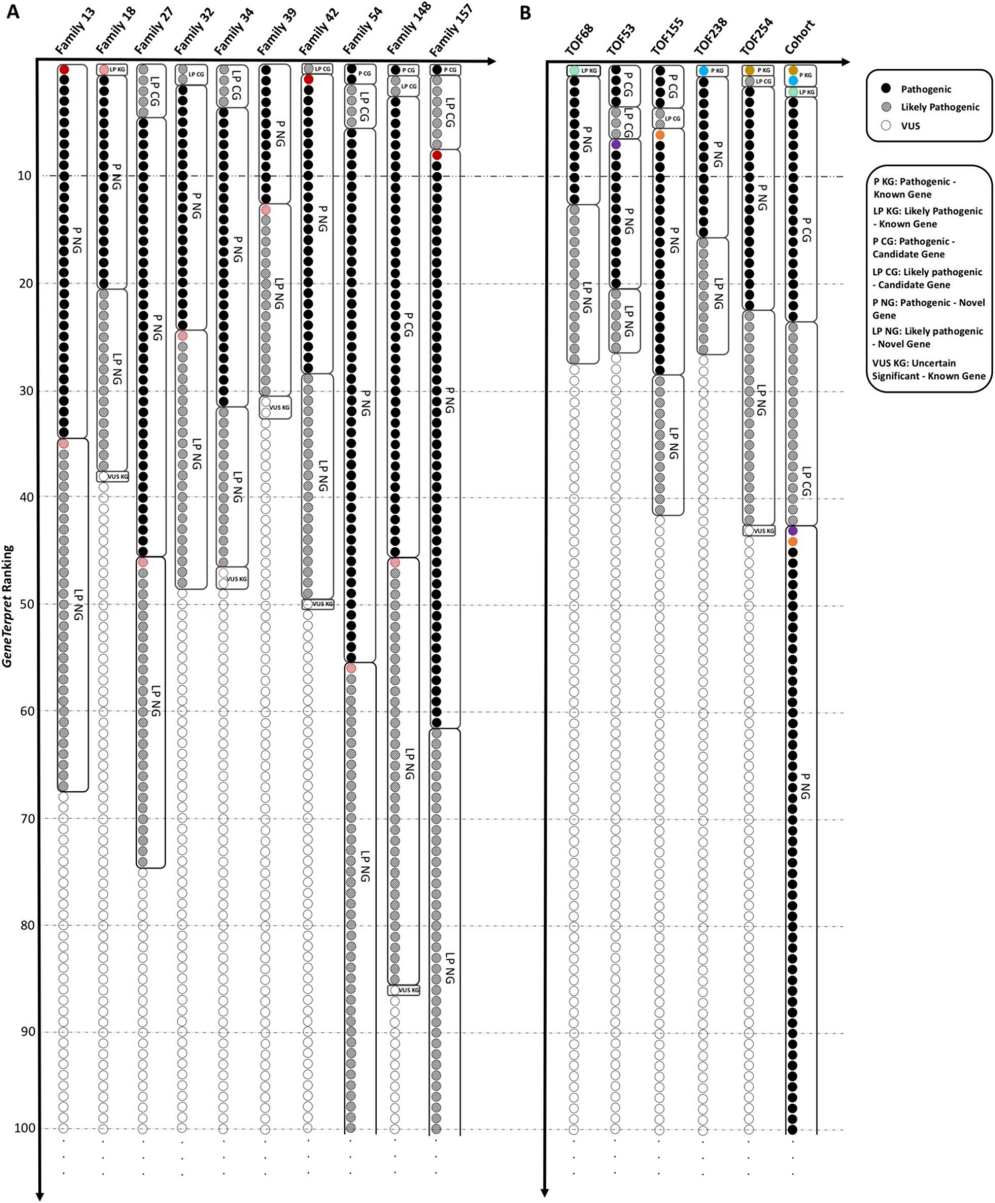
Graphical representation of the results from an analysis of internal datasets by *GeneTerpret* and manual interpretation. **A** The top hundred of ranked variants from the family-based analysis of ten families are represented. The red colour is highlighting the variant of interest (VOI) selected by a human analyst as published before [21]. The boxes around the variants cluster the same ranked variants by *GeneTerpret* (the same pathogenicity and validity terms). **B** The cohort-based results for 20 unrelated probands with “Tetralogy of Fallot”. The top hundred ranked variants are plotted as circles from top to bottom. The only five VOIs selected by a human genome analyst in five patients from this cohort [20] are highlighted in colours. Different colours have been selected to distinguish the VOI related to each patient. For comparison, individual analysis of genomes from the five probands with VOIs are also plotted using the same colour-coding. For instance, the purple colour represents the obtained VOI for patient TOF53 (one of the probands in the cohort). This variant is ranked 44 in the cohort-based analysis and ranked 8 in the singleton-based analysis by *GeneTerpret*

We tested 10 parent–child trios (VCF files) from a previous whole-genome sequencing (WGS) study of pediatric patients with cardiac phenotypes [22]. In 5 of 10 families, the variant of interest (VOI) identified through manual curation was ranked among the top 10 variants in *GeneTerpret*’s output. Expanding the list to the top 50 ranked variants led to the inclusion of 9 out of the 10 final calls by an expert geneticist interpretation. Notably, *GeneTerpret* correctly identified all de novo variants from the families tested. Overall, 8 out of 12 *VIP* classified pathogenic variants were in complete agreement with results from previous manual interpretation. Three of the variants not in concordance with the previous expert-review included a variant each in *NIPBL, PTEN*, and *MYH11* gene from family FAM32, FAM13, and FAM54 respectively. The other discordance variant also in FAM34 (previously classified as likely pathogenic by manual interpretation) was re-classified a VUS by *VIP*: FLT4 (NM_182925.4) c.89delC, p.(Pro30Argfs*3)—frameshift variant did not fulfill the PM2 category of being rare/absent in controls (minor allele frequency (MAF) of 5E-04 in gnomAD versus our stringently defined cut-off of MAF < 1E−5). Figure 2A summarizes *GeneTerpret*’s output in comparison with the previously interpreted variants*.* Diseases/phenotypes used as the input of gene-validity modules to generate gene validity scores for each family are listed in the Additional file 1: Table S7.

#### GeneTerpret’s interpretation performance in a cohort of individual samples

To assess *GeneTerpret’s* ability to process multiple VCF files from a cohort of individual samples, we analyzed a dataset containing 20 unrelated probands, including five that had VOI findings as published in a previous study of tetralogy of Fallot [21]. Figure 2B and Additional file 1: Table S8 summarize the results obtained from both the cohort-based and individual analysis. Notably, there was a complete agreement between *GeneTerpret’s* classification and the expert geneticist’s manual interpretation. Moreover, the VOI was always in the top 50 variants in *GeneTerpret’s* output (three out of five ranked within the top 10 ranked variants). *GeneTerpret* also classified additional variants as pathogenic, likely pathogenic in candidate and novel genes, and variants of unknown significance in candidate genes. Important to note that the clinical significance of these additional variants requires a review by an analyst and/or further lab investigation. They may contain potential secondary findings, additional causal variants or modifiers for the phenotype of interest.

The variants included in the study were run through Mutalyzer [24] to check their Human Genome Variation Society (HGVS) compliance (batch file generated is included as an Excel file—Additional file 3: Table S9).

#### GeneTerpret’s interpretation time

*GeneTerpret*’s use reduced the genome analysis and interpretation time from days/hours to minutes for a typical trio and from years/months to hours/minutes for a typical cohort of several hundred genomes. For instance, by inviting four genome scientists in our center, we shaped an internal assessment on the time required to generate narrowed lists of VOIs for the family-based analysis using *GeneTerpret* versus manual interpretation methods. The interpretation time by internal experts was reduced from an average of hours to a few minutes. In this assessment, at least 20 trio genomes were assigned to each of genome scientists. The time for investigating the single nucleotide variants in a family took on average 3–10 h, depending on the complexity of the case and genome interpretation skill of each of scientists. This included filtering and prioritization, and the application of the ACMG criteria to the top candidates. Integrating *GeneTerpret* into the analytic strategy reduced this time to an average of 15–20 min. Running *GeneTerpret* itself took only an average of 3–5 min per case. This internal assessment showed the interpretation time by experts was reduced from an average of hours to a few minutes.

## Discussion

*GeneTerpret* is a user-friendly visual analytics platform that utilizes information from a variety of databases and modules to assist speeding up the laborious process of genome variant interpretation (GVI). This platform was designed and implemented to streamline and optimize the expert genome analysis process by automating the data gathering, comparison, and filtration steps of GVI. To computationally achieve this, we re-packaged the data and computational tools into workable and tunable modules that can be connected to different pipeline networks through an intuitive graphical user interface (GUI). This GUI also allows the user to tailor the platform output by adjusting the connections between modules within the library to suit their needs. Although *GeneTerpret* helps to automate much of the GVI process, the user analysts remain in control, especially by providing gene-lists or tissue lists, adjusting the weight of each validity term, and performing the final ranked output review. The investigative process of connecting the pieces of evidence among seemingly disconnected modules reflects various strategies that different genome analysts employ to decipher the causal genes and organize the genomic variants based on phenotypic information.

*GeneTerpret* is encouragingly accurate when compared with expert-curated datasets in well-established public records of clinically relevant variants, such as DECIPHER and ClinGen. *GeneTerpret*’s *VIP* showed a high concordance (83.5%) in calling variant pathogenicity for the pathogenic or likely pathogenic variants in DECIPHER. When it comes to gene-disease validity, the *KING* module showed an extremely high agreement (97.2%) with ClinGen expert-curated table when identifying genes with moderate to strong evidence for 451 diseases.

*GeneTerpret* significantly facilitates genome interpretation. Based on the analysis of our internal and equally expert-reviewed data, the variants of interest were mostly ranked in the top 10 of *GeneTerpret*’s output (top 50 for all cases except one). It has the ability to efficiently analyze singletons, trios or cohorts by generating a manageable, prioritized list of variants for further in-depth interpretation. Even at the cohort level, *GeneTerpret* accelerated genome interpretation dramatically. Indeed, using *GeneTerpret* on a cohort of 30 samples took just a few minutes, generating a robust, manageable ranked list of variants. *GeneTerpret* would substantially reduce the time required to interpret large genomic datasets, particularly for large cohort analysis, which can take months to analyze.

*GeneTerpret* final ranking although affected by the set thresholds is generally accurate. For example, in the analyzed families the final clinically selected variants were often ranked within the top 50 by *GeneTerpret*. However, we caution that using the *GeneTerpret* platform does not prevent the need for a human interpreter to prevent potential misclassifications. Instead, it is a platform that offers an efficient aid to aggregate validity evidence and rank the variants, thus significantly reducing the time needed by an interpreter to sieve through an unsorted VCF file.

## Conclusions

A growing number of commercial or free packages are now used to aid with genome analysis. However, only a handful provide phenotype-driven variant prioritization. These tools are often too complex for routine use, force the users to accept and follow the designed routine, or give not enough or too many user-defined parameters (see Additional file 2: Table S1). Some are designed as black-box (closed-box) systems where the user is given minimal knowledge of the system architecture, and as a result, cannot gain access to the internal modules of the system. We do not claim that *GeneTerpret* addresses all these shortcomings or is superior to previous tools. Still, in a diverse world, we believe our platform provides significant improvement to what is available. A direct comparison of these platforms would be limited by their experimental approach, dependency on the human analyst, and lack of a standard “correct” output. At this stage, these tools primarily aid a clinical geneticist in sorting potentially interesting variants/genes. The potential relevant question is to survey analysts about their experience with various tools, which is beyond the scope of this manuscript.

We believe that an ideal genetic data analysis set of tools should be flexible, with multiple features under one platform as *GeneTerpret* is, and the associated tools should be designed as a white-box system for the users to see and interact with, allowing for full interactive information flow. However, we acknowledge that the implementation of a white-box system is complex and would be computationally impossible on a web-based platform. To balance the system’s accuracy, speed and efficiency, we developed *GeneTerpret* as a gray-box system, which balances the user’s engagement time and level of information. Our design attempts to not only allow users to understand the system and access the designed modules in the library, but also to provide a workspace environment to check the result of each module independently while showing the users which modules can be connected to make their interpretation routine meaningful.

Over time, more user preferences and analytical options will be included as computational abilities and technologies continue to advance. *GeneTerpret,* in its current version, has a few notable limitations. First, it is limited to analyzing single nucleotide variants (SNVs) with no functionality to analyze copy number or structural variations. Second, its functionality in analyzing familial data is limited to trios. Third, given the rigidity of some of the criteria (such as population frequency and haploinsufficiency cut-offs), the final call may differ from that conducted by an expert genetic variant interpreter (geneticist) who understands more nuanced scenarios. Finally, some of the parameters and filters for pathogenicity and validity are not customizable. We intend to provide more customization and interactive visual feedback in the future. *GeneTerpret* will make the genome analysis pipeline more streamlined and help to facilitate gene discovery. Importantly, *GeneTerpret* effectively addresses two main challenges: (1) it reduces the time of interpretation significantly by collecting evidence and sorting variants, and (2) it provides a visual, flexible workspace for the analyst to develop and customize their routine.

## Supplementary Information

**Additional file 1**: Materials file. **Table S2.** *GeneTerpret modules and respective databases with links to the used data.* **Table S3.** The required VCF file annotation, headers and descriptions. **Table S4**. The standard format for the PED file. **Table S5**. Variant Interpretation Program (*VIP*) logic (pseudocode) for variant classification following ACMG criteria. **Table S6.** Bins Used for Ranking *GeneTerpret* Output. **Table S7**. Comparison of *GeneTerpret* output and previous manual interpretation of 10 trios. **Table S8**. Comparison of *GeneTerpret* output and previous manual interpretation of a cohort with 20 TOF patients. Findings from manual interpretation have been reported for five individuals in this cohort.

**Additional file 2: Table S1.** Comparison of *GeneTerpret* and other GVI platforms

**Additional file 3: Table S9.** A list of variants included in the study as validated by the Mutalyzer [24]. The variants were checked to ensure their Human Genome Variation Society (HGVS) compliance.

**Additional file 4: Figure S1.** A snapshot of the *GeneTerpret* graphical user interface (*GeneTerpret GUI*). A general interpretation routine is depicted as an example. The user selects the needed modules from the top right panel; then drags and drops them one by one in the left workspace panel. Furthermore, the tissue or phenotype/disease of interest can be directly entered by the user as an input in the bottom right panel and the generated module could be dragged and dropped in the left workspace panel. The users can upload their annotated VCF file, gene list(s), family information (PED file) and phenotypes/diseases list as further input for *GeneTerpret* by tapping on the upload tab in the bottom right panel and drag and drop the assigned generated module for the uploaded file in the workspace panel in the left side.

**Additional file 5: Figure S2.** Gene Validity Module architecture. External databases are first fetched and filtered based on certain criteria, and the results are entered into MongoDB collections. *ExPhenosion*, *CanGene*, and *KING* modules take in user input and the MongoDB collections to perform their functions.

**Additional file 6: Figure S3.** Variant Interpretation Program (*VIP*) internal structure. External databases used for VIP are fetched and processed, with the output being stored in a MongoDB collection. In *VIP*, each database collection is associated with a specific *ACMG* classification, but not all classifications use these collections. Each row of the input VCF file is inputted to all classifications, flagging them as 1 or 0. Then, using the logic outlined in Supplementary Table S3, the individual classifications are combined to provide the pathogenicity classification for each variant.

**Additional file 7: Figure S4.** Overview of the causality module output; (A) the interactive visualization of variant distribution in the validity-pathogenicity space allows users to explore the desired variants. Dark green, light green, yellow, orange, and red colours represent the pathogenicity of variants in a 5-tier system: benign, likely benign, uncertain significance, likely pathogenic, and pathogenic variants. (B) Lasso filter allows the analyst to select the desired variants and filter them to a downloadable VCF file.

## Data Availability

*GeneTerpret* is a web-based interpretation tool available online at https://geneterpret.com/. Direct web links and databanks names corresponding to all of the datasets obtained from web-based sources used in our study are listed in the Additional file 1: Table S2.

## Abbreviations

ACMG: American College of Medical Genetics
GVI: Genomic Variant Interpretation
VCF: Variant Call Format
PED: Pedigree
GUI: Graphic User Interface
KING: Known INvolved Genes
ExPhenosion: Expanded Phenotype Exploration
HPO: Human Phenotype Ontology
CanGene: Candidate Genes
VIP: Variant Interpretation Program
VUS: Variant of Uncertain Significance
VOI: Variant Of Interest
KG: Known Gene
NG: Novel Gene
CG: Candidate Gene
WGS: Whole-Genome Sequencing
SNVs: Single Nucleotide Variants
