# Supplementary material for: GeneTerpret: a customizable multilayer approach to genomic variant prioritization and interpretation
Source: BMC Med Genomics. 2022 Feb 18;15:31. doi: 10.1186/s12920-022-01166-3 (PMC8857790; doi:10.1186/s12920-022-01166-3)
Supplement: Supplementary file 4 — Additional file 4: Figure S1. A snapshot of the GeneTerpret graphical user interface (GeneTerpret GUI). A general interpretation routine is depicted as an example. The user selects the needed modules from the top right panel; then drags and drops them one by one in the left workspace panel. Furthermore, the tissue or phenotype/disease of interest can be directly entered by the user as an input in the bottom right panel and the generated module could be dragged and dropped in the left workspace panel. The users can upload their annotated VCF file, gene list(s), family information (PED file) and phenotypes/diseases list as further input for GeneTerpret by tapping on the upload tab in the bottom right panel and drag and drop the assigned generated module for the uploaded file in the workspace panel in the left side. [file 12920_2022_1166_MOESM4_ESM.pdf]

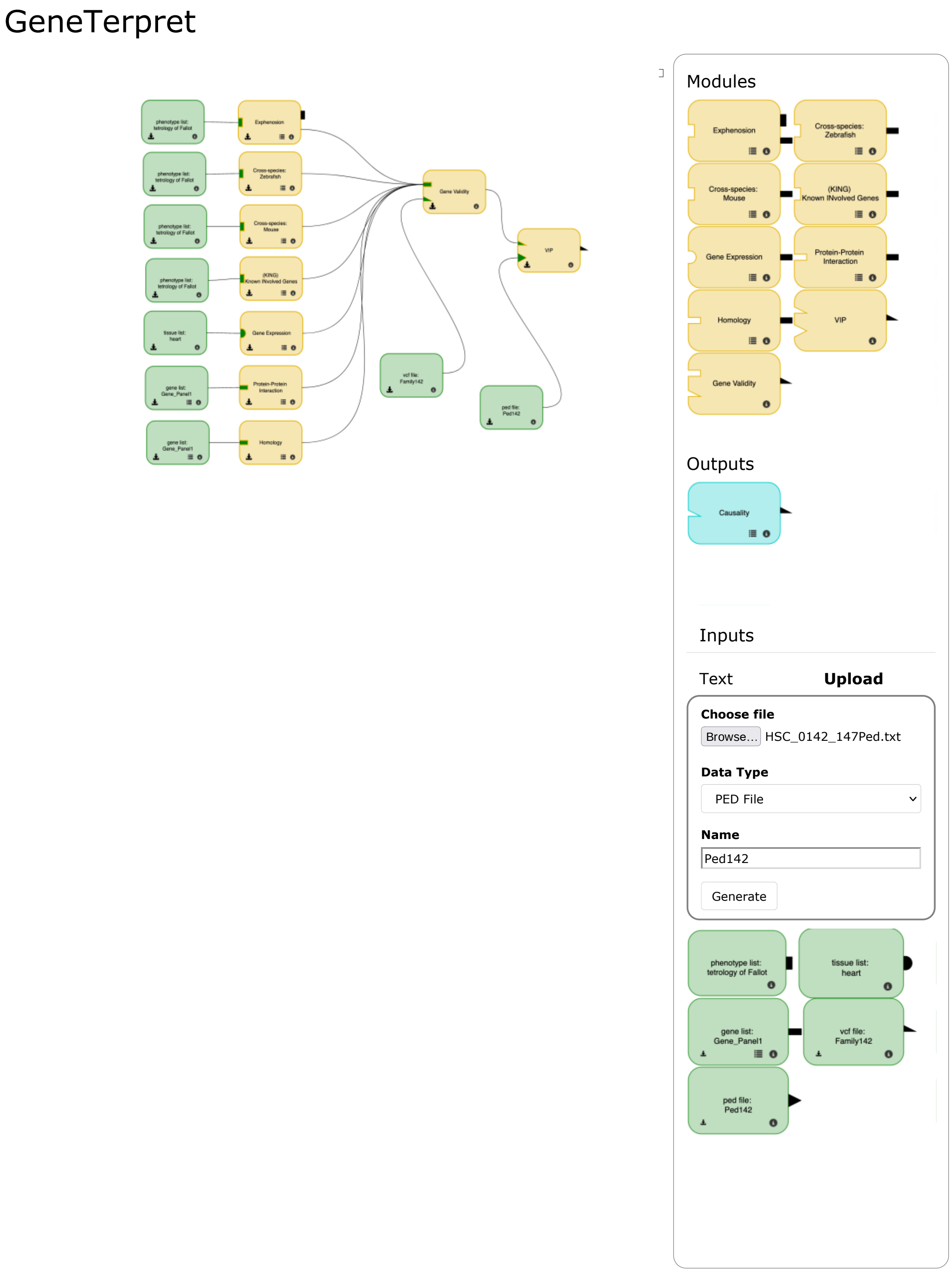

Supplementary Figure S1) A snapshot of the *GeneTerpret* graphical user interface (*GeneTerpret GUI*). A general interpretation routine is depicted as an example. The user selects the needed modules from the top right panel; then drags and drops them one by one in the left workspace panel. Furthermore, the tissue or phenotype/disease of interest can be directly entered by the user as an input in the bottom right panel and the generated module could be dragged and dropped in the left workspace panel. The users can upload their annotated VCF file, gene list(s), family information (PED file) and phenotypes/diseases list as further input for *GeneTerpret* by tapping on the upload tab in the bottom right panel and drag and drop the assigned generated module for the uploaded file in the workspace panel in the left side.
