# Supplementary material for: GeneTerpret: a customizable multilayer approach to genomic variant prioritization and interpretation
Source: BMC Med Genomics. 2022 Feb 18;15:31. doi: 10.1186/s12920-022-01166-3 (PMC8857790; doi:10.1186/s12920-022-01166-3)
Supplement: Supplementary file 5 — Additional file 5: Figure S2. Gene Validity Module architecture. External databases are first fetched and filtered based on certain criteria, and the results are entered into MongoDB collections. ExPhenosion, CanGene, and KING modules take in user input and the MongoDB collections to perform their functions. [file 12920_2022_1166_MOESM5_ESM.pdf]

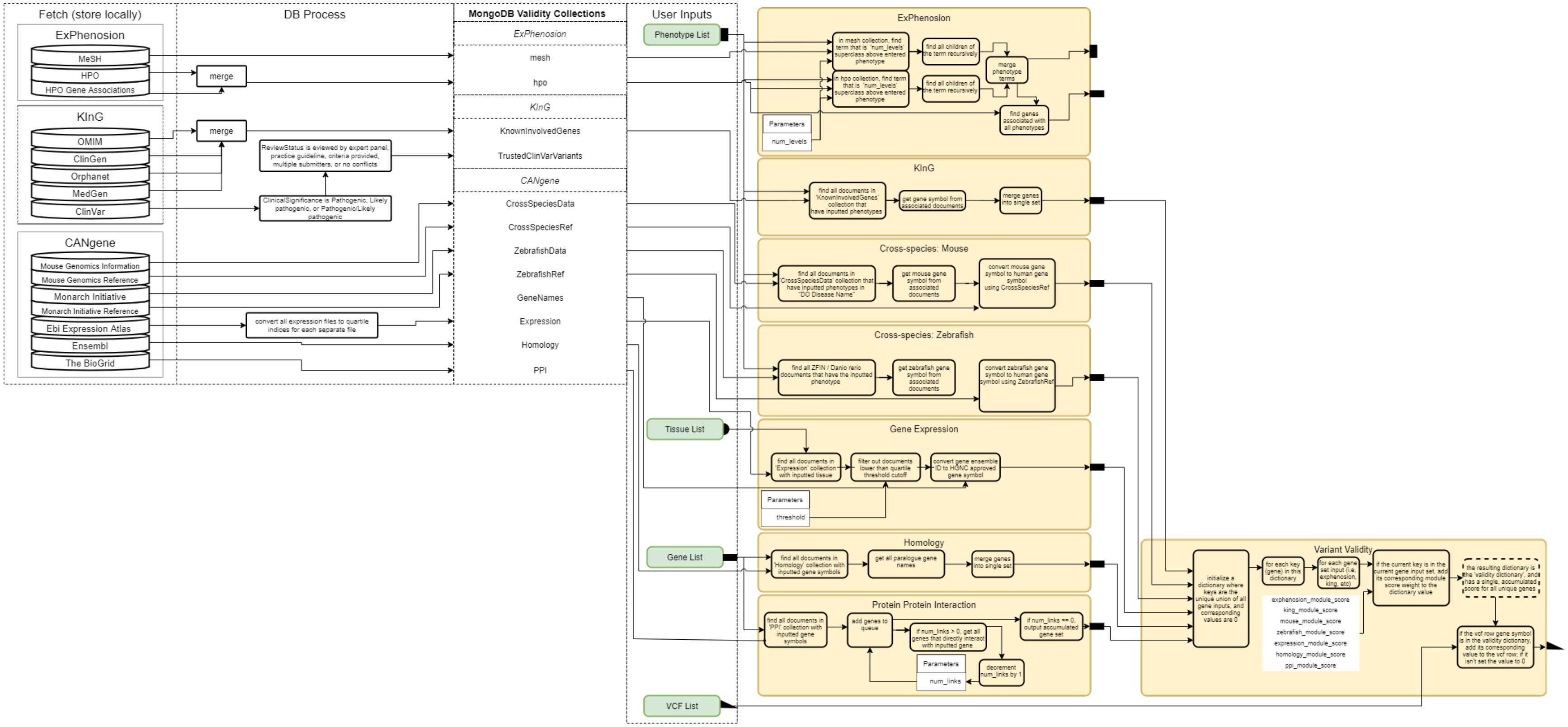

Supplementary Figure S2) Gene Validity Module architecture. External databases are first fetched and filtered based on certain criteria, and the results are entered into MongoDB collections. *ExPhenosion*, *CanGene*, and *KING* modules take in user input and the MongoDB collections to perform their functions.
