# Supplementary material for: GeneTerpret: a customizable multilayer approach to genomic variant prioritization and interpretation
Source: BMC Med Genomics. 2022 Feb 18;15:31. doi: 10.1186/s12920-022-01166-3 (PMC8857790; doi:10.1186/s12920-022-01166-3)
Supplement: Supplementary file 6 — Additional file 6: Figure S3. Variant Interpretation Program (VIP) internal structure. External databases used for VIP are fetched and processed, with the output being stored in a MongoDB collection. In VIP, each database collection is associated with a specific ACMG classification, but not all classifications use these collections. Each row of the input VCF file is inputted to all classifications, flagging them as 1 or 0. Then, using the logic outlined in Supplementary Table S3, the individual classifications are combined to provide the pathogenicity classification for each variant. [file 12920_2022_1166_MOESM6_ESM.pdf]

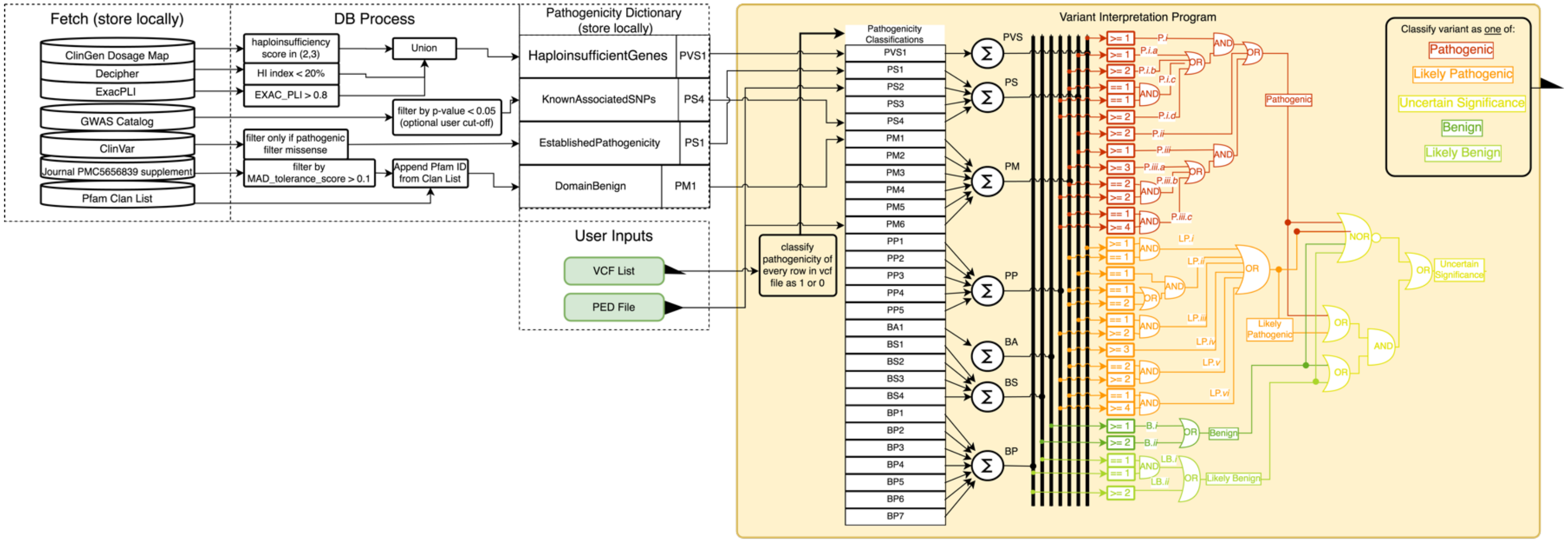

Supplementary Figure S3) Variant Interpretation Program (*VIP*) internal structure. External databases used for VIP are fetched and processed, with the output being stored in a MongoDB collection. In *VIP*, each database collection is associated with a specific *ACMG* classification, but not all classifications use these collections. Each row of the input VCF file is inputted to all classifications, flagging them as 1 or 0. Then, using the logic outlined in Table S2, the individual classifications are combined to provide the pathogenicity classification for each variant.
