# Supplementary material for: GeneTerpret: a customizable multilayer approach to genomic variant prioritization and interpretation
Source: BMC Med Genomics. 2022 Feb 18;15:31. doi: 10.1186/s12920-022-01166-3 (PMC8857790; doi:10.1186/s12920-022-01166-3)
Supplement: Supplementary file 7 — Additional file 7: Figure S4. Overview of the causality module output; (A) the interactive visualization of variant distribution in the validity-pathogenicity space allows users to explore the desired variants. Dark green, light green, yellow, orange, and red colours represent the pathogenicity of variants in a 5-tier system: benign, likely benign, uncertain significance, likely pathogenic, and pathogenic variants. (B) Lasso filter allows the analyst to select the desired variants and filter them to a downloadable VCF file. [file 12920_2022_1166_MOESM7_ESM.pdf]

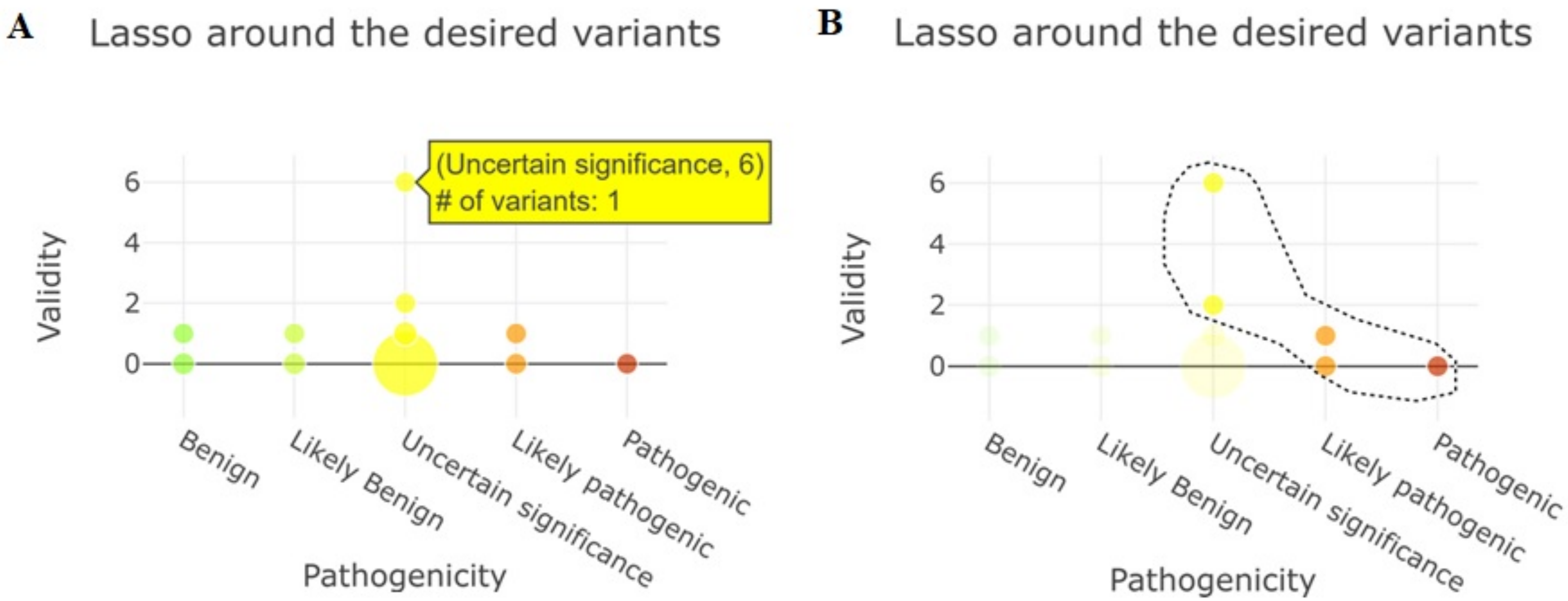

Supplementary Figure S4) Overview of the causality module output; **(A)** the interactive visualization of variant distribution in the validity-pathogenicity space allows users to explore the desired variants. Dark green, light green, yellow, orange, and red colours represent the pathogenicity of variants in a 5-tier system: benign, likely benign, uncertain significance, likely pathogenic, and pathogenic variants. **(B)** Lasso filter allows the analyst to select the desired variants and filter them to a downloadable VCF file.
